# Anti-Apolipoprotein A-1 IgG Levels Predict Coronary Artery Calcification in Obese but Otherwise Healthy Individuals

**DOI:** 10.1155/2012/243158

**Published:** 2012-11-07

**Authors:** Alessandra Quercioli, Fabrizio Montecucco, Katia Galan, Osman Ratib, Pascale Roux-Lombard, Sabrina Pagano, François Mach, Thomas H. Schindler, Nicolas Vuilleumier

**Affiliations:** ^1^Division of Cardiology, Department of Medical Specialties, Geneva University Hospitals and University of Geneva, 4 Rue Gabrielle Perret-Gentil, 1211 Geneva, Switzerland; ^2^First Clinic of Internal Medicine, Department of Internal Medicine, University of Genoa, 6 Viale Benedetto XV, 16100 Genoa, Italy; ^3^Division of Nuclear Medicine, Geneva University Hospitals, 4 Rue Gabrielle Perret-Gentil, 1211 Geneva, Switzerland; ^4^Division of Laboratory Medicine, Department of Genetics and Laboratory Medicine, Geneva University Hospitals, 4 Rue Gabrielle Perret-Gentil, 1211 Geneva, Switzerland

## Abstract

We aimed at determining whether anti-apolipoprotein (apo) A-1 IgG levels are independent predictors of coronary artery calcification (CAC) and coronary endothelial dysfunction in obese and nonobese subjects without cardiovascular disease. 48 nonobese and 43 obese subjects were included. CAC score was measured by thorax scanner and defined by an Agatston score > 0. Coronary endothelial dysfunction was determined by measuring myocardial blood flow responses to cold pressor test (CPT) on PET/CT. Serum anti-apoA-1 IgG levels were measured by ELISA. Prevalence of coronary calcification was similar between the two study groups, but the prevalence of coronary endothelial dysfunction was higher in obese subjects. Anti-apoA-1 IgG levels and positivity rate were higher in obese than in nonobese individuals. CAC score was higher in anti-apoA-1 IgG positive subjects. ROC analyses indicated that anti-apoA-1 IgG levels were significant predictors of CAC > 0, but not of coronary endothelial dysfunction with a negative predictive value of 94%. Anti-apoA-1 IgG positivity was associated with a 17-fold independent increased risk of CAC > 0. In conclusion, those preliminary results indicate that anti-apoA-1 IgG autoantibodies are raised in obese subjects and independently predict the presence of coronary calcification in this population but not the presence of coronary endothelial dysfunction.

## 1. Introduction

Despite significant progress related to evidence-based preventive and medical strategies, atherosclerosis-related cardiovascular diseases still account for the majority of morbidity and mortality in Western countries [1, 2]. Cardiovascular risk stratification mostly relies on the assessment of the traditional cardiovascular risk factors, allowing clinicians to derive different cardiovascular risk stratification tools, such as the widespread Framingham risk score (FRS) [1–4]. Nevertheless, the FRS predictive accuracy for major adverse cardiovascular event occurrence has been shown to be suboptimal, especially for screening purpose [5–8], and prompted the medical community to improve current cardiovascular risk stratification strategies.

Among emerging candidates, coronary artery calcium (CAC) scoring, using noncontrast computed tomography, has been shown to be a very promising screening tool to rule out both coronary artery disease and major adverse cardiovascular events in different populations, outperforming conventional cardiovascular risk stratification tools [9–13]. However, its widespread use as a screening tool is likely to be impeded by its financial costs and by the potential harmful effects related to radiation exposure. Numerous inflammatory biomarkers and autoantibodies [14–17], have also been evaluated as promising cardiovascular risk stratification candidates. Among the latter, auto-antibodies to apolipoprotein A-1 (anti-apoA-1 IgG) have been reported to be independent predictors of major adverse cardiovascular events after myocardial infarction (MI) and in patients suffering from rheumatoid arthritis [18, 19]. In rheumatoid arthritis and acute chest pain patients, anti-apoA-1 IgG was shown to be a promising biomarker providing significant incremental prognostic information to clinical scores, such as FRS or NSTEMI-TIMI score [20, 21]. Finally, in patients with carotid atherosclerosis, high circulating levels of those auto-antibodies were associated with increased plaque vulnerability [22].

Obesity is a pathological condition, which is raising public concern because of its association with an increased cardiovascular morbidity and mortality. Indeed, endothelial function, a functional precursor of the coronary artery disease process, is altered in subjects with increased body weight [23]. However, knowing whether anti-apoA-1 IgG could be associated to obesity and predict a pathological CAC score (as a surrogate marker of coronary artery disease) [12, 24] in low cardiovascular risk patients has never been evaluated. Therefore, we explored the potential association between serum anti-apoA-1 IgG levels and CAC score in obese and nonobese patients. Furthermore, because endothelial dysfunction represents an early functional stage of atherogenesis [25, 26], we also investigated the potential relationship between serum anti-apoA-1 IgG levels and coronary circulatory dysfunction (as determined by PET/CT coronary flow responses).

## 2. Methods

### 2.1. Study Population and Design

The current investigation follows as subanalysis of a previous published study [23, 27], targeted on the assessment of the relationship between body weight and coronary circulatory function in healthy individuals. Among the original 111 enrolled subjects, we included in the current analysis, 91 age-matched subjects with increasing body weight without evidence for cardiovascular diseases. The criteria for exclusion of 20 subjects from the published cohort were the lack of availability of the serum samples for anti-apoA-1 IgG measurement, which prevented us to consider all the participants to the original study. The actual study population was divided into 2 groups according to the body mass index (BMI): 48 nonobese subjects (BMI < 30 kg/m^2^) and 43 obese subjects (BMI ≥ 30 kg/m^2^), without known traditional cardiovascular risk factors (such as arterial hypertension, smoking, and diabetes mellitus). No study applicant was under statins, any cardiac or vasoactive medication nor had a history of variant angina, a family history of premature coronary artery disease, or clinically manifested cardiovascular, or any other systemic disease. Before inclusion in the cardiac perfusion assessment test, study participants underwent a complete visit including a physical examination, electrocardiogram, blood pressure measurements, and blood puncture in a fasting state. Following inclusion, each study participant underwent dual X-ray absorptiometry (DXA; Hologic QDR4500A) to measure body composition, total fat amount burden, and fat distribution as described previously [23, 24, 28]. Then, 13N-ammonia PET/CT measurements of myocardial blood flow (MBF) at rest and during vasomotor stress were performed in the morning in a fasting state to assess coronary circulatory function along with multidetector computed tomography (MDCT) CAC assessment. The study was approved by the University Hospitals of Geneva Institutional Review Board, and each participant signed the approved informed consent form. This study has been conducted in compliance with the Declaration of Helsinki.

### 2.2. Study Endpoints

Three predetermined endpoints were considered for this explorative study.

The primary endpoint was to test the ability of anti-apoA-1 IgG to predict the presence of any coronary artery calcification on chest PET-CT scanner, as described below. The secondary endpoint was to explore the predictive value of anti-apoA-1 IgG for coronary endothelial dysfunction, as explained below. The third endpoint was to compare anti-apoA-1 IgG levels between obese and nonobese subjects. All PET/CT data were assessed by two senior cardiologists blinded to the participants' biochemical data.

### 2.3. Assessment of Coronary Artery Calcification by Chest PET/CT

As first step of the perfusion assessment, 64-slice MDCT of the Biograph HiRez TruePoint PET/CT scanner (64-sliceBiograph HiRez TruePoint PET-CT scanner, Siemens, Erlangen, Germany) determined CAC score. The scanner was operated in the single slice mode with an image acquisition time of 100 ms and a section thickness of 3 mm. Prospective ECG-triggering was done at 55% of the R-R interval. Contiguous slices to the apex of the heart were obtained. CAC was considered present if three or more contiguous pixels with a signal intensity of >130 Hounsfield Unit were identified. The size of the lesion was automatically calculated, and the CAC was scored using the Agatston algorithm. The CAC was computed across all lesions denoted within the left main, LAD, LCx, and RCA, and the sum of all lesion scores yielded the total CAC score [29, 30]. As recommended, we used the CAC scoring system in a binary fashion (CAC present or absent) [13, 29]. Accordingly, any Agatston score above 0 was considered as a present CAC lesion [13, 29].

### 2.4. Assessment of Coronary Endothelial Function on 13N-Ammonia PET/CT

Myocardial perfusion was determined with 13N-ammonia PET/CT. We ruled out a hemodynamic significant CAD in all study participants by ascertaining the absence of abnormality and a homogeneous distribution of 13N-ammonia uptake on visual evaluation and polar map analysis, at rest and during vasomotor stress. After intravenous injection of *≈*500–550 MBq of 13N-ammonia (equivalent to 10–20 mCi) [30], serial transaxial emission images were recorded (12 images per frame of 10 s each, 3 frames of 20 s each, and 6 frames of 30 s each) with PET/CT, and time-activity curves from the first 12 dynamic frames (12 for 10 s each) by means of a 2-compartment tracer kinetic model were used to calculate regional and global myocardial blood flow (MBF) in mL/g/min with PMOD software package (version 2.8 PMOD Technologies Ltd., Zurich, Switzerland) [23]. 13N-ammonia PET/CT determined regional MBFs at rest, then during cold pressor testing (CPT) (reflecting predominantly endothelium-dependent vasomotion) and during pharmacologically induced hyperemia with standard infusion of dipyridamole (140 mg/kg/min) (reflecting predominantly endothelium-independent vasomotion) [31]. Mean MBF in mL/g/min was yielded by averaging regional MBF values from the three major coronary artery territories on the polar map. Heart rate, blood pressure, and a 12-lead electrocardiogram were recorded continuously during each MBF measurement. Rate-pressure product (RPP) was used as an index of myocardial workload conditions by averaging heart rate and systolic blood pressure during the first 2 min of each image acquisition. To account for possible variations in intracoronary perfusion pressure, an index of global coronary vascular resistance (CVR) was determined as the ratio of mean arterial blood pressure (mm Hg) to MBF (mL/g/min). The presence of coronary endothelial dysfunction was concluded when the ΔMBF to CPT value was below 0.3, as previously described [12, 23].

### 2.5. Biochemical Analyses

Blood chemistry included plasma glucose, haemoglobin A1c (HbA1_C_), insulin, total cholesterol, high density lipoprotein (HDL) and low-density lipoprotein (LDL) cholesterol, triglycerides levels, and high-sensitive C-reactive protein (hs-CRP). Assessment of insulin resistance using the Homeostasis Model Assessment (HOMA) was determined as previously described [2]. All analyses were performed on routine autoanalysers. LDL-cholesterol levels were derived from the conventional Friedwald equation.

Anti-apoA-1 IgG serum levels were measured as previously described [18–22]. Briefly, Maxi-Sorb plates (Nunc) were coated with purified, human-derived delipidated apoA-1 (20 *μ*g/mL; 50 *μ*L/well) for 1 hour at 37°C. After 3 washes with phosphate buffered saline (PBS)/2% bovine serum albumin (BSA; 100 *μ*L/well), all wells were blocked for 1 hour with 2% BSA at 37°C. Samples were diluted 1 : 50 in PBS/2% BSA and incubated for 60 minutes. Additional patient samples at the same dilution were also added to an uncoated well to assess individual nonspecific binding. After 6 further washes, 50 *μ*L/well of signal antibody (alkaline phosphatase-conjugated antihuman IgG; Sigma-Aldrich) diluted 1 : 1,000 in PBS/2% BSA solution was incubated for 1 hour at 37°C. After 6 more washes (150 *μ*L/well) with PBS/2% BSA solution, the phosphatase substrate p-nitrophenyl phosphate disodium (50 *μ*L/well; Sigma-Aldrich) dissolved in diethanolamine buffer (pH 9.8) was added. Each sample was tested in duplicate, and absorbance, determined as the optical density at 405 nm (OD405 nm), was determined after 20 minutes of incubation at 37°C (VersaMax, Molecular Devices). The corresponding nonspecific binding value was subtracted from the mean absorbance value for each sample. The positivity cutoff was predefined as previously validated and set at an OD value of 0.6 and 37% of the positive control value as described earlier [18–22]. OD values ranged from 0 to 1.78, and corresponding index values were between 0 and 99.2%.

### 2.6. Statistical Analyses

Analyses were performed using Statistica software (StatSoft, Tulsa, OK, USA). Fisher's bilateral exact test and Mann-Whitney *U*-test were used where appropriate. ROC analyses were performed using analyse-it software for Excel (Microsoft, Redmond, WA, USA) to (i) confirm the cut-off values prospectively chosen for anti-apoA-1 IgG and (ii) determine whether anti-apoA-1 IgG alone, and in combination with the FRS score could improve the area under the curve (AUC) for pathological CAC score and coronary artery endothelial dysfunction prediction. AUC comparisons were performed according to the nonparametric approach proposed by DeLong et al. [32]. Due to limited sample size, reclassifications statistics were not performed. Associations between anti-apoA-1 IgG levels and study endpoints are presented as the odds ratio (OR) and corresponding 95% confidence intervals (95%CI). Multivariable analyses with logistic regression were used to assess associations between variables. In this model, CAC > 0 and ΔMBF to CPT value was below 0.3 were set as dependent variables, and FRS [4] (allowing for adjustment for the global CV risk profile including age, gender and cholesterol level, blood pressure, diabetes, and smoking status within a single continuous variable) was set as the unique confounder because of the limited sample size. *P* value below 0.05 was considered as statistically significant.

## 3. Results

### 3.1. Clinical and Metabolic Characteristics of the Study Population

Baseline demographic characteristics of the study population are shown in Table 1.

As expected, significant differences between groups were observed for BMI, waist to hip ratio, amount of body fat (Table 1). Obese subjects had lower HDL levels, higher triglycerides, and hs-CRP levels when compared to nonobese subjects (Table 1). Considering the glycemic profile, HbA_1C_ and HOMA score were increased in obese subjects, who displayed a higher Framingham risk score than control subjects (Table 1). However, given the absence of known traditional cardiovascular risk factors, the median value of FRS remained very low in both groups (Table 1). Higher median anti-apoA-1 IgG levels were observed in obese subjects when compared to nonobese subjects (*P* = 0.0003), and anti-apoA-1 IgG values above the predefined cut-off (anti-apoA-1 IgG positivity) were retrieved only in obese subjects with a prevalence of 14% versus 0% in control group (*P* = 0.009; Table 1).

### 3.2. Coronary Artery Calcification and Coronary Endothelial Dysfunction of Study Population

CAC > 0 was present in 12 of the 91 subjects, with a mean of 3.88 ± 13.61 (median is 0) according to the Agatston score (Table 2), far below the lower limit of the first threshold for risk stratification (100–400), as recommended by the European Guidelines on Cardiovascular Disease Prevention in Clinical Practice [13]. No significant differences in the Agatston score were observed between the two study groups, but obese patients had an impaired endothelial coronary vasorelaxation in response to CPT (ΔMBF to CPT from rest), when compared to nonobese subjects (*P* < 0.0001; Table 2). Accordingly, the prevalence of coronary endothelial dysfunction (ΔMBF to CPT from rest <0.3) was higher in obese than in nonobese subjects (Table 2).

### 3.3. Hemodynamic Parameters during PET Examination and Myocardial Blood Flow

As shown in Table 2, hemodynamic conditions during PET examination were comparable in the two groups. At baseline, systolic blood pressure between groups was comparable, but heart rate and the resting rate pressure product (RPP) were higher in obese than in nonobese subjects (Table 2). Sympathetic stimulation with CPT induced an increase in RPP as compared to rest. This increase (ΔRPP), however, did not significantly differ between obese and nonobese subjects (Table 2), indicating comparable conditions of myocardial workload between the study groups. During pharmacologic vasodilation with dipyridamole, the change in RPP (ΔRPP) was also comparable between the 2 groups (Table 2).

The myocardial blood flow was significantly higher in obese than in nonobese subjects (Table 2). However, after adjustment for the RPP, the normalized MBF (NMBF) at rest was comparable in both groups. The ΔMBF to CPT and the NMBF during CPT significantly decreased between nonobese and obese subjects (Table 2). In order to account for possible variations in intracoronary driving pressure underlying the abnormal responses of the MBF, we calculated the cardiovascular resistance (CVR). This index significantly and inversely mirrored the alterations of MBF for the study groups (Table 2) ruling out possible interferences of the intra-aortic pressure on the results.

Dipyridamole-stimulated hyperaemic MBF was comparable between the 2 groups (Table 2). However, the total coronary vasodilator capacity (myocardial flow reserve [MFR]) significantly declined from nonobese to obese subjects.

### 3.4. FRS and Anti-Apoa-1 IgG Levels Predict the Presence of Coronary Artery Calcification

In nonobese subjects, receiving operator curves (ROC) analyses indicated that only FRS was found to be a good and significant predictor of CAC > 0, with an AUC of 0.83 (95% CI: 0.59–0.87; *P* = 0.0006), whereas anti-apoA-1 IgG and hs-CRP were not (Table 3). At the cut-off of 10%, the FRS had a sensitivity (SN) of 33% (95% CI: 4–78) and a specificity (SP) of 100% (95% CI: 92–100). Negative predictive values (NPV) and positive predictive values (PPV) for CAC > 0 prediction were of 91% (95% CI: 78–97) and 100% (95% CI: 20–100), respectively. Risk analyses could not be performed as only 2 patients had a FRS above 10%.

In obese subjects, ROC curve analyses indicated that anti-apoA-1 IgG was a significant predictor of a CAC > 0 with an AUC of 0.79 (95% CI: 0.54–1, *P* = 0.01) and FRS displayed an AUC of 0.85 (Table 3). On the other hand, hs-CRP, was not found to be a significant predictor of CAC > 0 (Table 3). Neither the AUC difference between anti-ApoA-1 IgG and FRS or between FRS and anti-apoA-1 IgG combined to FRS were found to be significant (*P* = 0.74 and *P* = 0.84, resp.).

At the cut-off of 10%, the FRS had a SN of 50% (95% CI: 12–88) and SP of 91% (95% CI: 77–98). NPV and PPV for CAC > 0 prediction were of 91% (95% CI: 76–98) and 50% (95% CI: 13–86), respectively. At the prespecified and previously validated anti-apoA-1 IgG cut-off, this test had a SN and SP of 60% (95% CI: 15–95) and 92% (95% CI: 78–98), respectively. NPV and PPV for CAC > 0 prediction were of 94% (95% CI: 79–99) and 50% (95% CI: 14–86), respectively.

Consistent with those results, obese patients tested positive for anti-apoA-1 IgG had higher median Agatston score than subjects tested negative for those antibodies (2.05 versus 0, *P* = 0.003; Table 4). There was also a significant correlation upon Spearman test between Agatston score and anti-apoA-1 IgG levels (*r* = 0.34, *P* = 0.03), and no correlation was retrieved between anti-apoA-1 IgG levels and FRS score (Spearman correlation: *r* = −0.05, *P* = 0.65). Accordingly, the presence of CAC > 0 was markedly higher in obese subjects tested positive for those auto-antibodies (67% versus 5%, *P* = 0.001, Table 4). Translated into risk analyses, anti-ApoA-1 IgG positivity increased the risk CAC > 0 by 17-fold (univariate odds ratio: 16.5, 95% CI: 1.9–140.8, *P* = 0.01) and result remained unchanged after the adjustment for the FRS risk score (OR: 52.96, 95% CI: 1.6–151.5; *P* = 0.02). On the other hand, having a FRS score above 10% increased the risk of a CAC lesion by 15-fold (odds ratio: 14.9, 95% CI: 2.58–86.3; *P* = 0.002), which remained unchanged after adding anti-apoA-1 IgG positivity to the model (odds ratio: 13.2, 95% CI: 1.71–101.2; *P* = 0.01). This indicates that both FRS and anti-apoA-1 IgG represent to distinct and independent predictors of CAC lesion, as already suggested by the absence of correlation between FRS and anti-apoA-1 IgG levels. In line with this hypothesis, combining anti-apoA-1 IgG positivity and having a FRS > 10% increased the risk of CAC > 0 to 24-fold (OR: 24.2; 95% CI: 2.4–245; *P* = 0.007).

In both nonobese and obese subjects, coronary endothelial function was not found to a be a significant predictor of the presence of CAC > 0 upon ROC curve analyses (AUC: 0.59; [95% CI: 0.36–0.83], *P* = 0.78, and AUC: 0.49 [95% CI: 0.29–0.69], *P* = 0.54, resp.), and no association was retrieved between the Agatston score and coronary endothelial dysfunction using Spearman correlation (and *r* = 0.004, *P* = 1 and *r* = 0.09; *P* = 0.75, resp.).

### 3.5. FRS is the Only Predictor of Coronary Endothelial Dysfunction

As shown in Table 3, FRS was found to be the only significant predictor of coronary endothelial dysfunction in both nonobese and obese subjects with respective AUC of 0.73 (95% CI: 0.59–0.87) and 0.87 (95% CI: 0.80–0.94), while serum anti-apoA-1 IgG levels and hs-CRP were not. For this reason, risk analyses for coronary endothelium dysfunction prediction were not performed for those circulating auto-antibodies and hs-CRP.

### 3.6. Anti-apoA-1 IgG Status and Lipid Profile

As shown in Table 4, subjects tested positive for anti-apoA-1 IgG had significant lower total cholesterol, triglycerides, and LDL cholesterol levels, whereas no difference was observed for HDL-cholesterol levels. Also, anti-apoA-1 IgG positive patients tended to have lower BMI and a better endothelial-dependent vasodilatory response to CPT. No significant differences nor trends were observed for the remaining parameters.

## 4. Discussion

The main finding of the present work is that high serum levels of anti-apoA-1 IgG are an independent predictor of calcified coronary artery lesions on chest computed tomography in obese but otherwise healthy subjects without any other cardiovascular risk factor. Those results extend previous works by indicating that anti-apoA-1 IgG positivity not only predict poor cardiovascular prognosis [18–21], but also the presence of coronary artery lesions, known to be a strong determinant of CV outcome in most populations studied [9–13]. Indeed, our results demonstrate that high levels of anti-apoA-1 IgG increased by 17-fold the risk of having a CAC score above 0, which remained unchanged after the adjustment of the FRS. Because safety and cost issues will be the major limiting factors to refrain from using CAC score as a widespread screening test for cardiovascular diseases in low risk populations [13], our results point to serum anti-apoA-1 IgG as a promising cardiovascular risk stratification tool candidate in obese patients, as suggested earlier [19–21]. On the other hand, hs-CRP did not appear useful for this purpose. Given the good negative predictive value (94%) of those auto-antibodies to predict the presence of a very minimal CAC score, our preliminary results indicate that in obese subjects, low anti-apoA-1 IgG levels could allow clinicians to identify very low risk patients who should avoid performing chest computed tomography for CAC score assessment. To this respect, knowing whether combining FRS to anti-apoA-1 IgG could provide incremental information to FRS alone, as suggested by the fact that the AUC increased from 0.85 for FRS alone to 0.88 (when combined to anti-apoA-1 IgG) and that OR raised from 16.5 to 24.2, represent an interesting option. Nevertheless, because of the nonsignificant nature of this increase according to Delong method, and to the fact that reclassification statistics could not be performed in the present study due to sample size limitations, this hypothesis needs to be challenged on bigger prospective trials.

On the other hand, anti-apoA-1 IgG serum levels were not found to be predictors of coronary endothelial dysfunction, which was surprising as this feature is well accepted to be one of earliest stage of atherogenesis [25]. This discrepancy can be due to the fact that although related to the same pathophysiological continuum, coronary artery calcification could involve distinct cellular mechanisms, as suggested by the absence of association between coronary endothelial function and the Agatston score retrieved in this study. Another point favoring this hypothesis is that anti-apoA-1 IgG has been so far mostly related to late rather than early stage of atherogenesis, by increasing atherosclerotic plaque vulnerability, with a less albeit relevant effect on atherogenesis [22]. Another likely explanation could be linked to power issues related to our limited sample size.

It is well known that obese subjects, even in the absence of other known cardiovascular risk factors, present an increased risk of cardiovascular morbidity and mortality [33]. The pathophysiological reasons underlying this observation are still poorly understood and could be related to BMI-related autoimmune-mediated processes, as overweight has been associated to an increased susceptibility of developing auto-immune diseases, potentially due to a leptin/adiponectin proinflammatory imbalance [33, 34]. The fact that the prevalence of serum anti-apoA-1 IgG positivity was raised in obese subjects to the same extend than what has been retrieved in other high cardiovascular risk settings [18–21], lend modest weight to this hypothesis and further links autoimmunity, cardiovascular diseases, and obesity. Surprisingly, obese patients tested positive for those auto-antibodies had a better lipid profile (lower total cholesterol, LDL cholesterol, and triglycerides) when compared to obese subjects tested negative for those auto-antibodies. Although highly speculative at the moment, those results further indicate that the association retrieved here between anti-apoA-1 IgG and a CAC > 0 is likely to be independent of lipid levels. On the other hand, because those auto-antibodies have been described to interfere with the anti-inflammatory and antioxidant functions of HDL [35, 36], which is known to be reduced in obese patients [37], we cannot exclude the fact that a loss in HDL function could contribute to the increased CAC score observed in obese patients with high serum anti-apoA-1 IgG levels.

Interestingly, our results demonstrated that those auto-antibodies were predictive of CAC > 0 only in obese subjects but not in patients with a BMI below 30. Among possible hypotheses, this observation can be related to the known overactivation of innate immune receptors, such as toll-like receptors (TLRs) occurring in obese patients [38, 39]. Because our previous work demonstrated that anti-apoA-1 IgG was promoting sterile inflammation through TLR2/CD14 complex [40], it is possible that in obese patients the anti-apoA-1 IgG-related TLR2 inflammation could be drastically facilitated, leading to enhanced coronary artery lesion development. This phenomenon could therefore explain why the anti-apoA-1 IgG association with CAC > 0 was found only in obese subjects and not in nonobese participants, but this speculative assumption needs experimental validation.

The major limitation of this hypothesis generating study is related to the very limited sample size due to stringent exclusions criteria, allowing us to demonstrate that in obese patients without cardiovascular diseases or other cardiovascular risk factors, anti-apoA-1 IgG is associated to obesity and predicts the presence of morphological coronary lesion. Despite this size limitation, the prevalence of high anti-apoA-1 IgG levels retrieved in the present study (14%) was of the same order of magnitude than what has been previously retrieved in other high cardiovascular risk populations [18, 19, 21, 22], suggesting that the occurrence of a selection bias is unlikely to have blunted the results of this study, although not formally excluded. Whether this predictive ability will persist in unselected populations with a higher cardiovascular risk burden is unknow, and restricts the scope of our conclusions to the population presently examined. Also, it can be argued that the burden of coronary calcification in the present study was too low to be clinically meaningful. This is easily explained by the fact that our cohort was composed by relatively young and healthy subjects without known cardiovascular diseases which, by definition, have a low likelihood of coronary artery disease and thus of advanced structural alterations. Nevertheless, as the aim of the present study was to test the ability of anti-apoA-1 IgG to exclude the presence of calcified coronary lesions for screening purposes, we believe that this aspect might be rather considered as strength than a weakness of the present study. Another important limitation of this work is that we could not conclude to the possible superiority of FRS over anti-apoA-1 IgG in this study. Nevertheless, even if this point needs to be resolved in further bigger prospective trials, our results suggest based upon spearman correlation and logistic regression that anti-apoA-1 IgG and FRS represent two distinct and independent predictors of coronary lesion on chest computed tomography.

## 5. Conclusions

This hypothesis generating study revealed that anti-apoA-1 IgG serum levels are associated to obesity and are independent predictors of CAC score >0 with a negative predictive value of 94%. For this reason, those preliminary results indicate that anti-apoA-1 IgG might be used as a promising cardiovascular screening tool in obese patients to identify low cardiovascular risk patients in whom coronary investigations could be overlooked. Those encouraging results need to be confirmed in larger prospective clinical studies before any clinical recommendation can be done.

## Figures and Tables

**Table 1:** Characteristics of the study population.

	Nonobese subjects	Obese subjects	*P* value
	(*n* = 48)	(*n* = 43)	
Age, yrs	42.5 (36–49)	44 (36–56)	0.36
Male Gender (%)	56 (27)	60 (26)	0.83
BMI, kg/m^2^	25.6 (22.1–27.3)	41.3 (31.8–45.0)	<0.0001
Waist, cm	92.3 (84.5–100.5)	117.5 (111–124)	<0.0001
Waist-hip ratio	0.88 (0.83–0.92)	0.96 (0.90–1.00)	<0.0001
Total fat amount, kg	16.7 (13.5–20.9)	42.3 (29.5–58.3)	<0.0001
Body fat, %	22.5 (19.0–27.8)	38.2 (30–46.7)	<0.0001
Cholesterol levels, mg/dL	195 (169.7–226.2)	195 (167.7–226.2)	0.81
LDL level, mg/dL	126.9 (110.8–147)	120.1 (102.2–143.9)	0.33
HDL level, mg/dL	48.8 (39.4–59.7)	42.3 (37.8–46.8)	0.01
Triglyceride level, mg/dL	74.3 (53.8–106.4)	122.4 (82.8–188.7)	<0.0001
Glucose level, mg/dL	94.6 (86.5–102.7)	99.5 (91.9–108.1)	0.01
Insulin, mUI/L	4.8 (1.9–7.8)	9.95 (6.4–19.5)	0.0001
HOMA	1.2 (0.5–1.8)	2.26 (1.65–4.60)	0.0001
Hs-CRP, mg/L	0.9 (0.9–2.9)	5.0 (2.9–8.5)	<0.0001
HbA_lc_, %	5.2 (5.05–5.4)	5.4 (5.2–5.7)	0.01
FRS, %	1 (0.9–3.0)	2 (0.9–5)	0.07
Anti-ApoA-1 IgG index	9.3 (2.4–20.5)	18.9 (11.4–25.3)	0.0003
Anti-ApoA-1 IgG positivity, % (*n*)	0 (0)	14 (6)	0.009

Values are medians (Interquartile Range (IQR)), unless indicated otherwise. BMI: body mass index; hs-CRP: high sensitive C-reactive protein; HbA_lc_: haemoglobin A_lc_; HDL: high-density lipoprotein; HOMA: Homeostasis Model Assessment; LDL: low-density lipoprotein; FRS: Framingham Risk Score. *P* values according to Mann-Whitney *U*-test for continuous values and according to bilateral exact Fisher test for proportions.

**Table 2:** Coronary artery calcification (CAC) score, myocardial blood flow (MBF) and hemodynamic parameters during PET studies.

	Nonobese subjects	Obese subjects	*P* value
	(n = 48)	(n = 43)	
Coronary calcification			
Agatston score	0 (0-0; 0–86.9)	0 (0-0; 0–77.1)	0.88
Subjects with CAC > 0, % (*n*)	13 (6)	14 (6)	1
Myocardial blood flow, MBF (mL/min/g) at rest			
MBF at rest	0.70 (0.63–0.76)	0.78 (0.66–0.90)	0.01
NMBF at rest	0.97 (0.85–1.12)	0.94 (0.86–1.13)	0.88
MBF during CPT	0.92 (0.80–1.06)	0.85 (0.75–0.95)	0.08
ΔMBF to CPT from rest	0.25 (0.16–0.31)	0.10 (0.03–0.16)	<0.0001
ΔMBF to CPT < 0.3, % (*n*)	73 (35)	93 (40)	0.01
NMBF during CPT	0.97 (0.88–1.14)	0.77 (0.69–0.91)	<0.0001
MBF during hyperemia	2.12 (1.79–2.47)	2.10 (1.72–2.42)	0.46
MFR	3.13 (2.57–3.60)	2.69 (2.21–3.30)	0.01
MBF hyperemia/NMBF rest	2.18 (1.86–2.64)	2.24 (2.0–2.14)	0.74
CVR (mmHg/mL/min/g)			
At rest	124.4 (107.8–137.0)	112.8 (97.1–134.3)	0.19
During CPT	104.9 (88.9–124.4)	128.2 (110–140)	0.001
Δ change to CPT from rest	−19.91((−31.4)–(−6.0))	5.81 (−5.6–17.1)	<0.0001
Pharmacologic vasodilation	37.9 (32.9–45.9)	42.7 (36.7–53.6)	0.03
Hemodynamics at rest			
Heart rate (beats/min)	62 (56.5–69)	66 (60–73)	0.01
Systolic blood pressure (mm Hg)	116.5 (110.5–126)	122 (110–132)	0.10
RPP	7090 (6321–7727)	7788 (7080–9000)	0.006
CPT			
Heart rate (beats/min)	70 (61.5–79)	73.5 (68–80)	0.01
Systolic blood pressure (mm Hg)	135 (126.5–146)	148 (131–157)	0.02
RPP	9300 (8165–10500)	10804 (9324–12480)	0.004
ΔRPP (CPT-rest)	2144 (1102–2837)	2745 (1228–3671)	0.20
Pharmacologic vasodilation			
Heart rate (beats/min)	85 (76.5–91)	87 (82–99)	0.05
Systolic blood pressure (mm Hg)	114 (109–121)	120 (115–130)	0.0005
RPP	9528 (8391–10556)	11171 (9914–12178)	<0.0001
ΔRPP (pharmacologic rest)	2273 (1498–3757)	2800 (2035–4283)	0.18

Values are median (Q1, Q3). MBF: myocardial blood flow; NMBF: normalized MBF; MFR: myocardial flow reserve; CVR: coronary vascular resistance; RPP: rate pressure products. *P* values according to Mann-Whitney *U* test for continuous values and to exact bilateral Fischer test for proportions.

**Table 3:** Respective predictive accuracies of anti-apoA-1 IgG and Framingham Risk Score (FRS) for the presence of CAC lesion and the presence of coronary endothelial dysfunction.

ROC curve analyses	Nonobese subjects (*n* = 48)	Obese subjects (*n* = 43)
	Area under the curve (95% CI), *P* value	Area under the curve (95% CI), *P* value
Abnormal coronary artery calcium score prediction (>0)		
Anti-apoA-1 IgG	0.54 (0.24–0.83), *P* = 0.41	0.79 (0.54–1.0), *P* = 0.01
FRS	0.83 (0.69–0.98), *P* < 0.0001	0.85 (0.68–100), *P* < 0.0001
Anti-apoA-1 IgG + FRS	0.79 (0.63–0.95), *P* = 0.0002	0.88 (0.70–100), *P* < 0.0001
hs-CRP	0.52 (0.28–0.76), *P* = 0.43	0.64 (0.36–0.92), *P* = 0.17
Coronary endothelial dysfunction prediction (ΔMBF during CPT < 30 mL/min/g)		
Anti-apoA-1 IgG	0.51 (0.31–0.70), *P* = 0.47	0.68 (0.10–100), *P* = 0.24
FRS	0.73 (0.59–0.87),* P* = 0.0006	0.87 (0.80–0.94), *P* < 0.0001
hs-CRP	0.53 (0.35–0.71), *P* = 0.36	0.60 (0.20–0.80), *P* = 0.30

**Table 4:** Clinical characteristics of obese subjects according to anti-apoA-1 IgG status.

	Obese Patients (*n* = 43)		
	Anti-apoA-1 IgG positive patients	Anti-apoA-1 IgG negative patients	*P* value
	(*n* = 6)	(*n* = 37)	
Age, years	47.5 (39–59)	43 (34.5–54)	0.23
Male gender; % (*n*)	67 (4)	57 (21)	1
Total Cholesterol, mg/dL	156.0 (124.8–175.5)	198.9 (175.5–230.1)	0.008
LDL Cholesterol, mg/dL	87.2 (70.2–113.1)	127.9 (102.6–150.2)	0.01
HDL Cholesterol, mg/dL	45.2 (43.7–46.0)	40.9 (37.4–46.8)	0.24
Triglycerides, mg/dL	62.7 (43.6–117.5)	124.6 (96.1–189.0)	0.01
Glucose, mg/dL	90.5 (86.5–108.1)	100.9 (91.9–108.1)	0.23
Insulin, mU/L	5.2 (5.2–8.3)	12.7 (7.3–21.6)	0.11
HOMA	1.22 (1.11–1.93)	3.59 (1.77–5.56)	0.15
HbA1C, %	5.4 (5.3–5.5)	5.4 (5.1–5.7)	0.92
Hs-CRP, mg/L	4.0 (1.5–13.1)	5.2 (2.9–8.5)	0.69
Framingham Risk Score (FRS)	2.5 (0.9–5)	2.0 (0.9–4)	0.80
BMI, kg/m^2^	32.2 (31.6–32.8)	42.0 (34.1–45.3)	0.08
Waist	116 (114–120)	119 (111–124)	0.86
Waist to hip ratio	0.98 (0.89–1.03)	0.96 (0.91–0.98)	0.53
Total fat, Kg	34.1 (27.3–39.9)	45.4 (32.9–58.6)	0.12
Percent of body fat	34.9 (24.8–40.5)	38.7 (30.1–47.9)	0.17
*Agatston score	2.05 (0–23.2; 0–38.3)	0(0-0;0–77.1)	0.003
Subjects with significant coronary lesion; % (*n*)	67 (4)	5 (2)	0.001
Myocardial blood flow (MBF);			
(mL/min/g):			
At rest	0.76 (0.55–0.79)	0.79 (0.66–0.93)	0.16
Normalized at rest	1.01(0.89–1.20)	0.92 (0.84–1.13)	0.42
During CPT	0.80 (0.71–1.13)	0.87 (0.76–0.95)	0.65
ΔMBF (CPT-rest)	0.16 (0.07–0.34)	0.09 (0.03–0.16)	0.16
Normalized during CPT	0.92 (0.81–1.21)	0.76 (0.67–0.88)	0.05
During hyperemia	2.12 (1.40–2.61)	2.11 (1.78–2.37)	0.85
Myocardial flow reserve; (mL/min/g)	2.92 (2.38–3.30)	2.62 (2.13–3.20)	0.62
MBF hyperemia/NMBF rest	2.14 (1.41–2.18)	2.13 (1.81–2.67)	0.56
Coronary vascular resistance			
(CVR); (mmHg/mL/mL/g)			
At rest	113.3 (90.3–132.1)	112.7 (97.1–134.3)	0.89
During CPT	119.7 (81.1–140)	128.2 (110.0–139.4)	0.49
ΔCVR (CPT-rest)	−3.25 (−9.17–7.95)	5.86 (−5.06–18.04)	0.59
In response to DP	40.6 (28.9–53.6)	42.8 (37.2–53.4)	0.17
Coronary endothelial dysfunction			
ΔMBF to CPT < 0.3, % (*n*)	67 (4)	97 (36)	0.05

Values are median (Q1, Q3), at the exception of *represented as (Q1–Q3; and range). *P* values according to Mann-Whitney *U* test for continuous values and to exact bilateral Fischer test for proportions.
